# Association Between Morning Blood Pressure Surge and Tinnitus in Hypertensive Patients: A Cross-Sectional Study

**DOI:** 10.3390/medicina62030509

**Published:** 2026-03-10

**Authors:** Nagehan Erdogmus Kucukcan, Abdullah Yildirim, Mustafa Lutfullah Ardic, Fadime Koca, Hakan Caf, Akif Kucukcan, Hasan Koca

**Affiliations:** 1 Department of Otorhinolaryngology, University of Health Sciences, Adana City Training and Research Hospital, 01230 Adana, Turkey; 2 Department of Cardiology, University of Health Sciences, Adana City Training and Research Hospital, 01230 Adana, Turkey; dr.yildirimabdullah@gmail.com (A.Y.); drmustafaardic@hotmail.com (M.L.A.); hasankoca90@hotmail.com (H.K.); 3 Department of Cardiology, Cukurova State Hospital, 01230 Adana, Turkey; drfadimekoca@gmail.com (F.K.); hakancaf@gmail.com (H.C.); 4 Laboratory of Microbiology, Cukurova State Hospital, 01230 Adana, Turkey; akifkucukcan@gmail.com

**Keywords:** tinnitus, morning blood pressure surge, hypertension, arterial stiffness, ambulatory blood pressure monitoring

## Abstract

*Background and Objectives*: Despite extensive research into its vascular mechanisms, the relationship between tinnitus and morning blood pressure surge (MBPS) remains unexplored. This study aims to investigate the association between tinnitus and MBPS in hypertensive patients. *Materials and Methods*: The study included 266 hypertensive patients, 86 with tinnitus and 180 without. Office blood pressure (BP) measurements, 24 h ambulatory BP monitoring (ABPM), echocardiographic findings, and laboratory parameters were analyzed. Tinnitus severity was assessed using the Tinnitus Handicap Inventory (THI). MBPS was calculated as the difference between the average systolic BP (SBP) in the first two hours after waking and the lowest three SBP values measured during sleep. Statistical analyses included regression models, ROC curve analysis, and the Boruta feature selection method. *Results:* MBPS was significantly higher in the tinnitus group compared to the non-tinnitus group (35 ± 9 vs. 26 ± 11 mm Hg, *p* < 0.001). Office BP and ABPM were significantly lower in the tinnitus group, while DBP showed no differences. The regression analysis identified MBPS (OR = 1.15, 95% CI: 1.08–1.23, *p* < 0.001), SBP (OR = 1.09, 95% CI: 1.03–1.15, *p* = 0.004), age (OR = 0.89, 95% CI: 0.82–0.96, *p* = 0.003), and smoking status (OR = 3.54, 95% CI: 1.09–11.61, *p* = 0.037) as independent predictors of tinnitus. The ROC analysis demonstrated that MBPS >28 mm Hg predicted tinnitus with 73.3% sensitivity and 68.3% specificity (AUC = 0.742, 95% CI: 0.685–0.793, *p* < 0.001). The comparative analysis showed that MBPS had a superior predictive accuracy for tinnitus compared to other BP parameters (*p* < 0.001). The 5-fold cross-validated ROC analysis further validated the moderate discriminatory power of MBPS, with an average AUC of 0.735 (95% CI: 0.672–0.798). *Conclusions:* This study demonstrates a significant association between tinnitus and MBPS in hypertensive patients. MBPS may serve as a useful indicator for identifying patients at risk of tinnitus, highlighting the importance of circadian BP monitoring in clinical practice.

## 1. Introduction

Many people may experience temporary “ear noises,” often described as whistling sounds, which may be associated with sudden or progressive hearing loss [1]. Tinnitus, defined as the perception of noise without any external stimulus, can affect one or both ears [2]. While generally temporary and benign, pathological tinnitus is considered to last longer than 5 min [3]. Its prevalence ranges from 10% to 15% in adults, increases with age, and significantly impacts quality of life [4].

Although the exact etiology of tinnitus remains unclear, it has been associated with genetic, vascular, neurological, and drug-related causes [5,6]. Among these, vascular factors such as carotid artery atherosclerosis, systemic arterial hypertension (SAH), and diabetes mellitus are frequently emphasized [7,8]. The relationship between SAH and tinnitus has been widely studied, but findings remain inconsistent. While some studies suggest a positive correlation between tinnitus and SAH, especially with advancing age, others have found no significant association. A systematic meta-analysis further highlighted contradictory findings regarding the relationship between SAH and tinnitus [8,9,10,11]. These discrepancies may be explained by differences in study populations, comorbidities, antihypertensive treatments, and the increased prevalence of hypertension with age [8,11,12].

Circadian blood pressure (BP) changes, characterized by a decrease during sleep and an increase upon awakening, are associated with a higher frequency of cardiovascular events in the morning [13,14]. Poor morning BP control, particularly in hypertensive patients, significantly increases cardiovascular risks [15]. Morning blood pressure surge (MBPS), a key parameter reflecting these fluctuations, has been identified as an important indicator of arterial stiffness (AS) and autonomic function in hypertensive patients [16,17]. Several vascular mechanisms may explain the association between MBPS and tinnitus. MBPS reflects early-morning sympathetic activation and increased arterial stiffness, which can impair cochlear microcirculation or induce turbulent blood flow, particularly in the carotid arteries. These hemodynamic disturbances may lead to vascular-related tinnitus, especially in individuals with underlying SAH [8]. Moreover, MBPS is associated with endothelial dysfunction and microvascular changes, both of which have been implicated in the pathogenesis of tinnitus [8,11]. Although vascular mechanisms underlying tinnitus have been extensively studied, the precise association between tinnitus and MBPS has not yet been thoroughly investigated. This study aims to examine the relationship between tinnitus and MBPS in hypertensive patients.

## 2. Materials and Methods

### 2.1. Study Design and Setting

This cross-sectional, single-center study included 266 consecutive patients with a history of hypertension who were admitted to the University of Health Sciences Adana City Training and Research Hospital between April and September 2021. Patients with tinnitus complaints lasting at least 3 months were categorized into the tinnitus group, while those without tinnitus were placed in the non-tinnitus group. All patients underwent physical, otological, and cardiovascular examinations. Weight, body mass index, pulse examinations, detailed medical history, drug use histories, and laboratory examinations were recorded. Transthoracic echocardiography was performed in the left lateral decubitus position using the Acuson SC2000 PRIME device and 4V1c probe (Siemens Healthineers, Erlangen, Germany), with images obtained during breath-holding and ECG integration. Left ventricular ejection fraction, left ventricular diameters, and left atrium diameters were measured and recorded.

Exclusion criteria were defined as patients with a history of otological disorders and hearing loss episodes, patients with tinnitus before the diagnosis of hypertension, uncontrolled or secondary hypertension, patients with hypertensive end-organ damage, patients with suspected white coat hypertension, and patients with advanced renal, hepatic and heart failure. Patients with a previous diagnosis of hypertension, currently receiving antihypertensive therapy, and normal physical examination and office BP measurements were not excluded.

The trial was approved by the University of Health Sciences Adana City Training and Research Hospital Clinical Research Committee. The study sites were in compliance with the Declaration of Helsinki (revised in 2013), and approval from the ethics committees and local authorities was obtained (approval number: 91-1611).

### 2.2. Tinnitus Diagnosis and Severity and Tinnitus Handicap Index

Patients with tinnitus were questioned about its duration, frequency, period, and location. Patients with complaints of tinnitus lasting at least 3 months were included. The severity of tinnitus was classified according to the Turkish version of the Tinnitus Handicap Inventory (THI). This version of the THI is a measure that is suitable, reliable, and valid for evaluating tinnitus degree in Turkish patients. To a total of 25 questions, patients answered “yes”, “sometimes”, or “no” (“yes” = 4 points, “sometimes” = 2 points, “no” = 0 point, totaling up to 100 points). Patients who received 0–16 points were classified as *slight*, 18–36 points as *mild*, 25–36 points as *moderate*, 37–48 points as *severe*, 49–60 points as *catastrophic* [18].

### 2.3. Office Blood Pressure Measurement

Office BP measurement was performed on both arms by a blinded cardiologist using a mercury sphygmomanometer (Rudolf Riester GmbH & Co. KG, Jungingen, Germany), with the appropriate cuff (12–13 cm wide and 35 cm long), and with the patients resting for at least 15 min, holding their arm at heart level, and not speaking. Korotkoff phases I and V (decrease/disappearance) were taken as reference to determine systolic blood pressure (SBP) and diastolic blood pressure (DBP). The BP was recorded by taking the average of three measurements made at 5 min intervals on the arm with the highest BP. All BP measurements adhered to the recommendations of the American Hypertension Society [19].

### 2.4. Ambulatory Blood Pressure Measurement

The Holter device was attached to all patients in the morning hours after the clinical examination, and 24 h ambulatory blood pressure monitoring (ABPM) was performed using whichever arm was used to measure the BP of the patients, with the appropriate position and the appropriate cuff, by means of a portable digital recorder device (GE Cardiosoft Tonoport V, Berlin, Germany). It was programmed to perform a measurement every 15 min between 07:00 a.m. and 11:00 p.m. and every 30 min between 11:00 p.m. and 07:00 a.m. The device settings were changed and recorded in accordance with the information obtained from the patient regarding their sleep and awaking times. The hours between falling asleep and waking up were considered night, and the other hours were considered daytime. In total, ABPM of the patients was performed for at least 24 h. Patients were single-blinded to the BP measurements. The records of the patients whose measurements were adequate at least 80% of the time were evaluated.

### 2.5. Morning Blood Pressure Surge

Various methods are available to detect MBPS with ABPM [20]. We chose the most commonly used method in this study. Using this method, we recorded the difference between the mean SBP value in the first 2 h after waking up and the mean of the lowest 3 SBP values measured during sleep.

### 2.6. Statistical Interpretation

**Figure 1:**
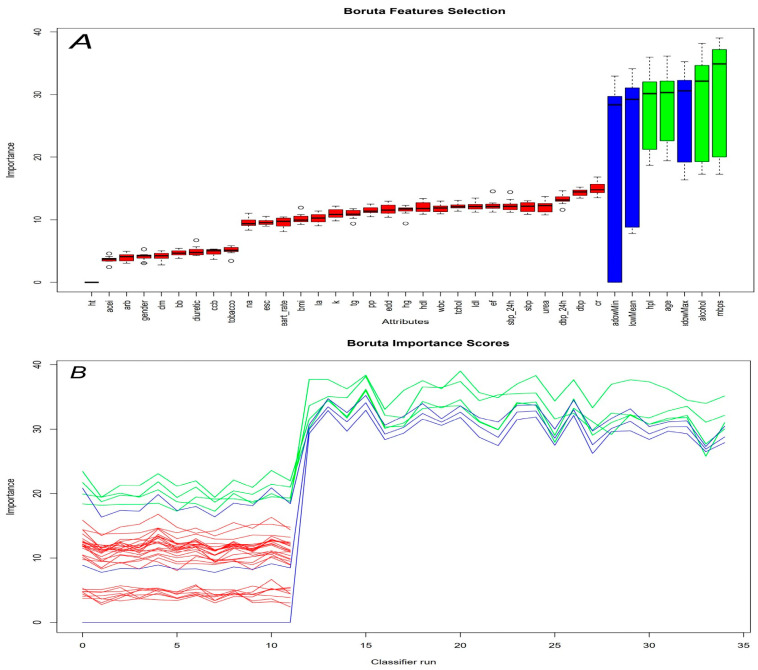
Feature selection for potential confounders associated with tinnitus using the Boruta algorithm. (**A**) The feature selection process. (**B**) The evolution of Z-scores during the screening process. The horizontal axis displays the variable names and the number of iterations.

Statistical analyses were performed using R software version 4.4.2 (Vienna, Austria; URL: http://www.R-project.org). Distribution analysis of continuous variables was conducted using histogram graphs, skewness, and kurtosis values, and the Kolmogorov–Smirnov test was used to determine normality. Normally distributed variables were expressed as mean ± SD. The Spearman correlation test was used for correlation analysis. The parameters most associated with tinnitus were identified using the Boruta selection method. Confirmed attributes (MBPS, alcohol use, age, and history of hyperlipidemia) with a Z-value higher than the ShadowMax feature were included in further adjusted analyses (Figure 1A,B). Regression analyses provided odds ratios (ORs) and 95% confidence intervals (CIs). Model performance metrics included the Brier score (lower values indicating better calibration), Akaike Information Criteria (lower values indicating better fit), adjusted R^2^ (higher values indicating better fit), and areas under the curve (AUC; <0.50 indicating no discrimination, >0.75 indicating good discrimination). The receiver operating characteristic (ROC) curves and AUCs were analyzed to assess the discriminative accuracy of MBPS, SBP, DBP, 24 h DBP, and 24 h SBP. The predictive performance of MBPS in tinnitus detection was evaluated using a k-fold cross-validation approach. This method divided the dataset into five subsets, iteratively training the model on four folds and testing it on the remaining fold. ROC curve analysis was performed for each fold, and AUC values were calculated. The average 5-fold AUC, along with its 95% CI, represented the overall model performance. Statistical comparisons of AUCs were conducted using the DeLong method to ensure robust evaluation of diagnostic accuracy. The optimal cutoff value for MBPS was determined using the maximum Youden index. All statistical analyses utilized two-sided tests with a significance level (alpha) of 0.05.

## 3. Results

**Figure 2:**
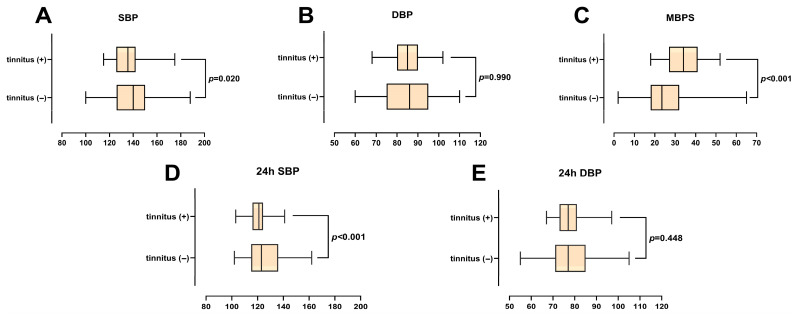
Boxplots display SBP, DBP, 24 h SBP, 24 h DBP, and MBPS between tinnitus-positive and tinnitus-negative groups. Significant differences are marked with *p* values. Abbreviations: DBP: diastolic blood pressure, MBPS: morning blood pressure surge, SBP: systolic blood pressure.

**Figure 3:**
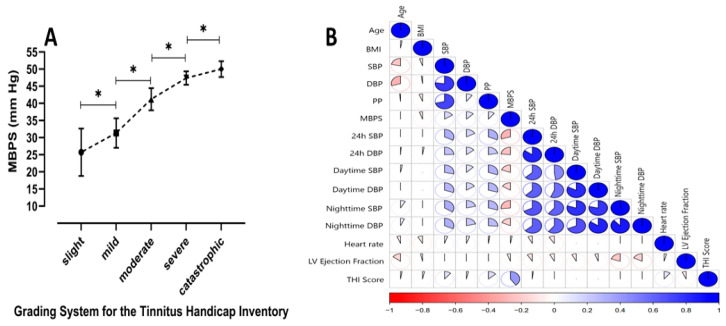
(**A**) Morning blood pressure surge values are shown across different Tinnitus Handicap Inventory grades. Error bars represent standard deviations, and significant differences between groups are marked with asterisks (*). * *p*-value < 0.05. (**B**) The matrix shows the pairwise correlations among variables such as BMI, MBPS, blood pressure parameters, and Tinnitus Handicap Inventory grade. Positive correlations are shown in blue, and negative correlations are in red, with the intensity indicating correlation strength.

A total of 266 patients with (n = 86) and without (n = 180) tinnitus were included in this study. The baseline demographic characteristics of the patients, such as age, gender, risk factors, and the treatments they received, are given in Table 1. In the group without tinnitus, the mean age was 54.5 ± 9.7 years and 55.6% were female; in the tinnitus group, the mean age was 55.3 ± 10.1 and 50.0% of the patients were female (*p* = 0.568, *p* = 0.395, respectively). In the office BP measurements, SBP was significantly lower in the tinnitus group compared to the non-tinnitus group (136 ± 13 vs. 141 ± 18 mm Hg, *p* = 0.020), while DBP showed no difference (84 ± 8 vs. 84 ± 13 mm Hg, *p* = 0.990). In the 24 h ambulatory measurements, SBP (121 ± 7 vs. 126 ± 14 mm Hg, *p* < 0.001) was lower in the tinnitus group, whereas DBP did not differ significantly (77 ± 5 vs. 78 ± 11 mm Hg, *p* = 0.448). MBPS was significantly higher in patients with tinnitus (35 ± 9 vs. 26 ± 11 mm Hg, *p* < 0.001) (Figure 2). There was no difference in antihypertensive treatments between the groups, except for diuretic use, which was higher, and calcium channel blocker use, which was lower in the tinnitus group (*p* = 0.019 and *p* = 0.038, respectively). In the analysis of laboratory parameters, hemoglobin and LDL-C levels were significantly lower in the tinnitus group (*p* = 0.003, *p* < 0.001, respectively), while urea levels were significantly higher (*p* = 0.008). Transthoracic echocardiographic findings showed similar left ventricular ejection fraction values between the two groups, but the end-diastolic diameter was significantly smaller in the tinnitus group (*p* = 0.015) (Table 1). The MBPS showed a statistically significant increase across the grading levels of the THI (slight, mild, moderate, severe, and catastrophic) (*p* < 0.001, Figure 3A). No significant correlation was identified between MBPS and other BP parameters (Figure 3B).

Binary logistic regression analysis was performed using predictors identified through the Boruta (age, MBPS, history of hyperlipidemia, and alcohol use) and univariable regression analyses (SBP, smoker, hemoglobin, urea, and diuretic use). In the multivariable model (performance parameters: adjusted R^2^ = 0.59, Harrell’s C statistic = 0.907, Brier score = 0.086, Akaike Information Criteria = 122.18), age (OR = 0.89, 95% CI: 0.82–0.96, *p* = 0.003), SBP (OR = 1.09, 95% CI: 1.03–1.15, *p* = 0.004), MBPS (OR = 1.15, 95% CI: 1.08–1.23, *p* < 0.001), and smoking status (OR = 3.54, 95% CI: 1.09–11.61, *p* = 0.037) were identified as independent predictors of tinnitus among hypertensive patients (Table 2).

**Figure 4:**
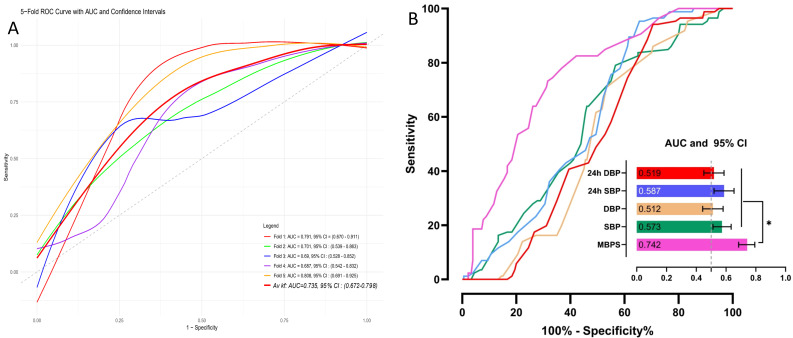
(**A**) The plot displays ROC curves for each fold in a 5-fold cross-validation, showing model performance in predicting tinnitus. The average k-fold AUC with a 95% CI is also included for overall evaluation. (**B**) ROC curves compare the predictive accuracy of different blood pressure parameters, including 24 h DBP, 24 h SBP, in-office DBP, SBP, and MBPS. AUC values with 95% CI are included. Analysis conducted using the DeLong method. * *p*-value < 0.001. Abbreviations: AUC: area under the curve, CI: confidence interval, DBP: diastolic blood pressure, MBPS: morning blood pressure surge, ROC: receiver operating characteristic, SBP: systolic blood pressure.

The 5-fold cross-validated ROC curves, where the average AUC for MBPS was calculated as 0.735 (95% CI: 0.672–0.798), indicating moderate discriminative power across the folds (Figure 4A). A comparative analysis of MBPS, SBP, and other BP parameters was conducted, showing that MBPS > 28 mm Hg (according to the maximal Youden index, Supplementary Figure S1) was a significant predictor of tinnitus with 73.3% sensitivity and 68.3% specificity (AUC: 0.742, 95% CI: 0.685–0.793, *p* < 0.001). Additionally, a statistically significant difference was observed in favor of MBPS compared to SBP, DBP, 24 h SBP, and 24 h DBP in predicting tinnitus, as indicated by the comparison analysis (all *p* < 0.001) (Figure 4B).

## 4. Discussion

This study demonstrated that MBPS was significantly higher in patients with tinnitus compared to those without and was closely linked to tinnitus severity, as measured by the THI. The logistic regression analysis identified MBPS, along with SBP, smoking status, and age, as independent predictors of tinnitus in hypertensive patients. MBPS also showed better predictive power for tinnitus than other BP parameters, with a threshold of >28 mm Hg providing high sensitivity and specificity. These findings suggest that MBPS could serve as a useful biomarker for evaluating tinnitus risk and severity.

The relationship between tinnitus and SAH has been repeatedly discussed in the literature. The results of scientific research and meta-analysis on this subject are also contradictory. Figueiredo et al. reported that there is a positive correlation between tinnitus and SAH, and this relationship gets stronger with increasing age [8]. In the subgroup analyses of the ELSA-Brasil registry study, which included 901 patients in whom auditory tests were performed, no association was found between SAH and tinnitus [9]. In a meta-analysis including 20 articles and 40,381 patients, a clear relationship between SAH and tinnitus could not be demonstrated, and the findings were reported to be contradictory [11]. Our findings also support this contradiction. In our study, we found lower office SBP in the tinnitus group.

The relationship between AS and tinnitus has been studied in previous articles. AS is a closely related but different concept to SAH that has gained importance in recent years. The main task of the arterial system is to transform the pulsatility caused by left ventricular ejection into a continuous flow, to transmit it to the periphery and to ensure the continuity of blood [21]. AS is closely related to atherosclerotic processes and occurs as a result of abnormal distribution of elastin and collagen in the vessel wall, the thickening of the arterial wall, and loss of elasticity [22,23]. The increase in AS is associated with decreased elasticity of the aorta and increased mechanical wall tension. These processes are closely related to the pathophysiology of vascular disease and atherosclerosis. Various methods are available for the assessment of AS. Today, they are the most preferred non-invasive methods. Pulse wave velocity (PWV) measurement from the radial, brachial, or carotid arteries comes first [24]. Cross-sectional population-based studies have reported that increased PWV and AS affect the brain and renal microcirculation, leading to lacunar infarction and kidney damage [25,26]. An analysis involving 443 healthy individuals also reported the association of PWV with silent cerebral hemorrhages and AS [27]. Gedikli et al. reported that there was no difference in SBP between the tinnitus and control groups, but that central aortic pressures and pulse wave velocities from the brachial artery were higher in the tinnitus group as indicators of AS, and these parameters were independent determinants [28]. However, besides the fact that measuring stiffness with this method requires a separate device and cuff, the technique has its own significant disadvantages [29]. Another way to evaluate aortic flexibility is ultrasonographic methods. They are based on direct measurements of the descending and ascending aorta by transthoracic and transesophageal echocardiography and a series of offline calculations. Kaneko et al. compared classical aortic stiffness parameters with aortic arch flexibility and aortic arch lateral wall velocities in ischemic stroke patients and found that they were more closely associated with ischemic stroke [30]. Yildirim et al. also examined AS in aortic stenosis patients with similar methods [31]. The most important disadvantage of this approach based on direct aortic imaging of aortic stiffness is that it is laborious. Considering these limitations in our research, we used MBPS, which is easy, reliable, reproducible, reflects BP fluctuation, and can be used as a stiffness parameter.

Morning hours are important for the biological body. Changes in body position and perception with awakening cause changes in the functioning of biological mechanisms together with the circadian system. As a result of these changes, the autonomic nervous system causes an increase in sympathetic activity, which directly affects the cardiovascular system and causes an increase in BP. This is the basic mechanism underlying MBPS. The adrenal system, which starts from the suprachiasmatic nucleus of the pituitary, which controls the circadian rhythm of the cardiovascular system, causes MBPS through the renin–angiotensin–aldosterone system and the sympathetic nervous system. Endothelial dysfunction, atherosclerotic processes, arterial remodeling, BP variability, baroreflex dysfunction, and AS are important determinants of this fluctuation [32]. An increase in MBPS is associated with an increased risk of cardiovascular disease; changes in vessels, such as atherosclerosis; inflammatory conditions; and small vessel disease. The relationship between MBPS and organ damage and cerebrovascular complications has been demonstrated in previous studies. MBPS has been shown to be a risk factor for cardiovascular disease and associated with vascular remodeling [33]. Its association with cardiovascular events, including cerebrovascular events, is known [34].

An important finding of the present study is the graded increase in MBPS across increasing THI levels. This observation suggests that MBPS may be associated not only with the presence of tinnitus but also with its clinical severity. As THI reflects the functional and emotional burden of tinnitus, the progressive elevation of MBPS across THI grades may indicate a link between exaggerated circadian BP variability and the degree of tinnitus-related distress [35]. This relationship supports the hypothesis that vascular and autonomic dysregulation, reflected by an excessive MBPS, may contribute to more severe tinnitus manifestations [8,11,35]. It is also possible that the relationship between MBPS and tinnitus severity is bidirectional. Higher levels of tinnitus-related distress may increase sympathetic activity through stress-related hormonal responses, including catecholamine and cortisol release, which can transiently elevate blood pressure and contribute to an exaggerated MBPS. Therefore, elevated MBPS may reflect not only vascular mechanisms contributing to tinnitus severity, but also the physiological consequences of stress associated with more severe tinnitus [36,37]. Such findings support the possibility of bidirectional interactions between stress and tinnitus severity.

There are different mechanisms that can explain the relationship between MBPS and tinnitus. The first of these may be related to ideal blood flow in vessels. The critical Reynolds (Re) number associated with the transformation of flow from laminar to turbulent in the vascular bed is related to flow velocity, blood viscosity, and vessel diameter [38]. If there is a change in these and the Re number decreases (e.g., carotid artery stenosis), turbulent flow will occur instead of laminar flow. It is known that if these changes occur in the carotid artery, the turbulent flow that occurs can lead to tinnitus [39]. These flow changes in the vascular bed, increased AS, and microvascular changes constitute similar mechanisms between MBPS and tinnitus. In addition, although we could not find patients with tinnitus as an independent predictor in our study, low hemoglobin values may have caused tinnitus by similar mechanisms. Low viscosity due to anemia may have caused flow changes in the partially obstructed or stenotic segments and, consequently, caused tinnitus [40]. The relationship between hemodynamic changes due to anemia and tinnitus has already been reported in various studies [41,42,43].

In our study, we examined patients diagnosed with SAH and receiving treatment. Thus, we eliminated the suspicion of pseudo-hypertension and the limitations of this situation. We also excluded patients with uncontrolled hypertension and associated end-organ damage, because it is clear that severe uncontrolled hypertension may have effects on cochlear microcirculation and may result in otological problems such as hearing loss and sensorineural tinnitus [44,45]. This situation revealed an important advantage for our study.

As a result, the relationship between SAH and tinnitus remains unclear, with conflicting findings reported in different patient populations and meta-analyses [8,9,10,11,12]. These findings suggest that SAH alone may not be sufficient to explain the mechanisms underlying tinnitus. To date, the relationship between tinnitus and MBPS has not been previously investigated. In this study, we evaluated hypertensive patients with and without tinnitus to examine the association between tinnitus and MBPS using ABPM. Our results showed a significant association between tinnitus and higher MBPS in patients with diagnosed hypertension. However, several limitations should be considered. First, the cross-sectional and single-center design limits causal interpretation and may reduce the generalizability of the findings. Second, although all participants had confirmed hypertension and were receiving antihypertensive treatment, medication use was analyzed only by drug class. Information on dose, timing, duration of use, and potentially ototoxic agents was not available, which may have influenced both MBPS and tinnitus [46]. In addition, tinnitus assessment was based on self-reported symptoms and the THI, without objective audiological testing or differentiation between tinnitus subtypes. This may have limited the interpretation of the underlying mechanisms and may partly explain the moderate predictive performance of the model. Residual confounding due to unmeasured factors such as psychological status, sleep disorders, and environmental or occupational noise exposure cannot be excluded. Therefore, although MBPS was higher in patients with tinnitus, it remains unclear whether this finding reflects a contributing vascular mechanism, tinnitus-related autonomic dysregulation, or an interaction of both. Future multicenter, prospective studies with more detailed clinical, pharmacological, and audiological assessments are needed to clarify these relationships.

## 5. Conclusions

In this study, we have shown that AS may be high in patients diagnosed with hypertension who have complaints of tinnitus. It should be noted that high BP fluctuation may occur even when office BPs are regulated to prevent mortality and morbidity in these patients.

## Figures and Tables

**Table 1:** Baseline traits of study population.

Variable	Non-Tinnitus Group (n = 180)	Tinnitus Group (n = 86)	*p*-Value *
Basic characteristics and admission parameters			
Age, years	54.5 ± 9.7	55.3 ± 10.1	0.568
Gender, female, n (%)	100 (55.6)	43 (50.0)	0.395
BMI, kg/m^2^	25.9 ± 3.4	25.5 ± 3.0	0.349
Alcohol use, n (%)	8 (10.0)	8 (9.3)	0.879
Diabetes mellitus, n (%)	55 (30.6)	29 (33.7)	0.603
Hyperlipidemia, n (%)	29 (16.1)	33 (38.4)	0.036
Smoker, n (%)	53 (29.4)	34 (39.5)	0.101
Weight, kg	72.1 ± 10	71.0 ± 11.8	0.521
In-office SBP, mm Hg	141 ± 18	136 ± 13	0.020
In-office DBP, mm Hg	84 ± 13	84 ± 8	0.990
Office heart rate, bpm	77 ± 12	79 ± 11	0.188
MBPS, mm Hg	26 ± 11	35 ± 9	<0.001
Average 24 h SBP, mm Hg	126 ± 14	121 ± 7	<0.001
Average 24 h DBP, mm Hg	78 ± 11	77 ± 5	0.448
Echocardiographic Parameters			
LV ejection fraction, %	63 ± 4	62 ± 5	0.231
LV end-diastolic diameter, mm	50 ± 10	48 ± 5	0.015
Left atrium diameter, mm	35 ± 4	34 ± 4	0.179
Laboratory Parameters			
White blood cell, ×10^3^/uL	7.93 ± 1.95	8.61 ± 2.38	0.023
Hemoglobin, g/dL	13.6 ± 1.9	13.0 ± 1.4	0.003
Urea, mg/dL	29.2 ± 8.9	31.9 ± 6.8	0.008
Creatinine, mg/dL	0.74 ± 0.14	0.84 ± 0.16	<0.001
Sodium, mEq/L	139 ± 3	138 ± 4	0.801
Potassium, mEq/L	4.4 ± 0.5	4.4 ± 0.5	0.255
LDL-C, mg/dL	124 ± 38	108 ± 31	<0.001
HDL-C, mg/dL	43 ± 14	42 ± 16	0.600
Triglycerides, mg/dL	152 ± 65	143 ± 68	0.331
Medications, n (%)			
ACEi	81 (45)	40 (46.5)	0.817
ARB	38 (21.1)	18 (20.9)	0.973
Beta-blocker	27 (15.0)	20 (23.3)	0.099
Diuretic	59 (32.8)	41 (47.7)	0.019
CCB	60 (33.3)	18 (20.9)	0.038

Data are shown as n (%), and mean ± standard deviation. * A *p*-value of <0.05 was considered statistically significant. Abbreviations: ACEi, angiotensin-converting-enzyme inhibitors; ARB, angiotensin receptor blockers; BMI, body mass index; CCB, calcium channel blocker; DBP, diastolic blood pressure; HDL-C, high-density lipoprotein cholesterol; LDL-C, low-density lipoprotein cholesterol; LV, left ventricular; MBPS, morning blood pressure surge; SBP, systolic blood pressure.

**Table 2:** Covariate and multivariable regression analyses to predict tinnitus.

	Covariate			Model ^&^		
	OR	95% CI	*p*-Value *	OR	95% CI	*p*-Value *
Age, *increase from 25 to 76 years*	1.00	0.98–1.03	0.567	0.89	0.82–0.96	0.003
Systolic blood pressure, *from 100 to 188 mm Hg*	0.98	0.97–1.00	0.035	1.09	1.03–1.15	0.004
MBPS, *from 2 to 65 mm Hg*	1.08	1.05–1.11	<0.001	1.15	1.08–1.23	<0.001
Smoker, *yes*	1.57	0.92–2.69	0.102	3.54	1.09–11.61	0.037
Hyperlipidemia, *yes*	2.11	1.05–4.29	0.037	1.26	0.42–3.82	0.676
Alcohol use, *yes*	1.08	0.39–3.04	0.879	5.23	0.77–35.29	0.089
Hemoglobin, *from 9.4 to 11.30 g/dL*	0.82	0.71–0.95	0.008	0.96	0.69–1.34	0.960
Urea, *from 11.3 to 59 mg/dL*	1.04	1.00–1.07	0.017	1.05	0.99–1.11	0.138
Diuretic use, *yes*	1.87	1.11–3.16	0.020	0.61	0.21–1.71	0.349

* *p* value < 0.05 was considered statistically significant. ^&^ Model performance parameters: adjusted R^2^ = 0.59, Harrell’s C statistic = 0.907, Brier score = 0.086, AIC = 122.18. Abbreviations: CI, confidence interval; OR, odds ratio.

## Data Availability

Data are available on request due to privacy and ethical restrictions.

## Supplementary Materials

The following supporting information can be downloaded at https://www.mdpi.com/article/10.3390/medicina62030509/s1, Figure S1: Youden index analysis used to determine the optimal MBPS threshold for predicting tinnitus.

## References

[1] Kiang N., Moxon E., Levine R. (1970). Sensorineural hearing loss. Ciba Foundation Symposium.

[2] Henry J.A., Dennis K.C., Schechter M.A. (2005). General review of tinnitus. J. Speech Lang. Hear. Res..

[3] Coles R. (1984). Epidemiology of tinnitus: (1) prevalence. J. Laryngol. Otol..

[4] Snow J.B. (2004). Tinnitus: Theory and Management.

[5] Seidman M.D., Standring R.T., Dornhoffer J.L. (2010). Tinnitus: Current understanding and contemporary management. Curr. Opin. Otolaryngol. Head Neck Surg..

[6] Altissimi G., Colizza A., Cianfrone G., de Vincentiis M., Greco A., Taurone S., Musacchio A., Ciofalo A., Turchetta R., Angeletti D. (2020). Drugs inducing hearing loss, tinnitus, dizziness and vertigo: An updated guide. Eur. Rev. Med. Pharmacol. Sci..

[7] Crummer R.W., Hassan G. (2004). Diagnostic approach to tinnitus. Am. Fam. Physician.

[8] Figueiredo R.R., Azevedo A.A., Penido N.D.O. (2016). Positive association between tinnitus and arterial hypertension. Front. Neurol..

[9] Samelli A.G., Santos I.S., Padilha F.Y.O.M.M., Gomes R.F., Moreira R.R., Rabelo C.M., Matas C.G., Bensenor I.M., Lotufo P.A. (2021). Hearing loss, tinnitus, and hypertension: Analysis of the baseline data from the Brazilian Longitudinal Study of Adult Health (ELSA-Brasil). Clinics.

[10] Yang P., Ma W., Zheng Y., Yang H., Lin H. (2015). A systematic review and meta-analysis on the association between hypertension and tinnitus. Int. J. Hypertens..

[11] Figueiredo R.R., de Azevedo A.A., Penido N.d.O. (2015). Tinnitus and arterial hypertension: A systematic review. Eur. Arch. Oto-Rhino-Laryngol..

[12] Borghi C., Brandolini C., Prandin M.G., Dormi A., Modugno G.C., Pirodda A. (2005). Prevalence of tinnitus in patients withhypertension and the impact of different anti hypertensive drugs on the incidence of tinnitus: A prospective, single-blind, observational study. Curr. Ther. Res..

[13] Muller J.E., Tofler G., Stone P. (1989). Circadian variation and triggers of onset of acute cardiovascular disease. Circulation.

[14] Elliott W.J. (1998). Circadian variation in the timing of stroke onset: A meta-analysis. Stroke.

[15] Redon J., Bilo G., Parati G., SURGE Steering Committee (2012). Home blood pressure control is low during the critical morning hours in patients with hypertension: The SURGE observational study. Fam. Pract..

[16] Okada Y., Galbreath M.M., Shibata S., Jarvis S.S., Bivens T.B., Vongpatanasin W., Levine B.D., Fu Q. (2013). Morning blood pressure surge is associated with arterial stiffness and sympathetic baroreflex sensitivity in hypertensive seniors. Am. J. Physiol.-Heart Circ. Physiol..

[17] Marfella R., Siniscalchi M., Nappo F., Gualdiero P., Esposito K., Sasso F.C., Cacciapuoti F., Di Filippo C., Rossi F., D’Amico M. (2005). Regression of carotid atherosclerosis by control of morning blood pressure peak in newly diagnosed hypertensive patients. Am. J. Hypertens..

[18] Newman C.W., Jacobson G.P., Spitzer J.B. (1996). Development of the Tinnitus Handicap Inventory. Arch. Otolaryngol.–Head Neck Surg..

[19] Pickering T.G., Hall J.E., Appel L.J., Falkner B.E., Graves J., Hill M.N., Jones D.W., Kurtz T., Sheps S.G., Roccella E.J. (2005). Recommendations for blood pressure measurement in humans and experimental animals: Part 1: Blood pressure measurement in humans: A statement for professionals from the Subcommittee of Professional and Public Education of the American Heart Association Council on High Blood Pressure Research. Circulation.

[20] Amodeo C., Guimarães G.G., Picotti J.C., dos Santos C.C., Fonseca K.D.B., Matins R.F., Cordeiro A.C., Sousa A.G.M.R. (2014). Morning blood pressure surge is associated with death in hypertensive patients. Blood Press. Monit..

[21] Vlachopoulos C., Alexopoulos N., Stefanadis C. (2010). Aortic stiffness: Prime time for integration into clinical practice. Hell. J. Cardiol..

[22] Selwaness M., van Den Bouwhuijsen Q., Mattace-Raso F.U., Verwoert G.C., Hofman A., Franco O.H., Van Der Lugt A., Witteman J., Wentzel J.J. (2014). Arterial stiffness is associated with carotid intraplaque hemorrhage in the general population: The Rotterdam study. Arterioscler. Thromb. Vasc. Biol..

[23] Zieman S.J., Melenovsky V., Kass D.A. (2005). Mechanisms, pathophysiology, and therapy of arterial stiffness. Arterioscler. Thromb. Vasc. Biol..

[24] Luzardo L., Lujambio I., Sottolano M., da Rosa A., Thijs L., Noboa O., A Staessen J., Boggia J. (2012). 24-h ambulatory recording of aortic pulse wave velocity and central systolic augmentation: A feasibility study. Hypertens. Res..

[25] Hashimoto J., Ito S. (2009). Some mechanical aspects of arterial aging: Physiological overview based on pulse wave analysis. Ther. Adv. Cardiovasc. Dis..

[26] Ishikawa T., Hashimoto J., Morito R.H., Hanazawa T., Aikawa T., Hara A., Shintani Y., Metoki H., Inoue R., Asayama K. (2008). Association of microalbuminuria with brachial–ankle pulse wave velocity: The Ohasama study. Am. J. Hypertens..

[27] Rogowicz-Frontczak A., Araszkiewicz A., Pilacinski S., Zozulinska-Ziolkiewicz D., Wykretowicz A., Wierusz-Wysocka B. (2012). Carotid intima-media thickness and arterial stiffness in type 1 diabetic patients with and without microangiopathy. Arch. Med. Sci. AMS.

[28] Gedikli Ö., Kemal O., Yıldırım U., Çeçen A.B., Karabulut H., Akcay M., Terzi O. (2020). Is there an association between the parameters of arterial stiffness and tinnitus?. Acta Oto-Laryngol..

[29] Segers P., Kips J., Trachet B., Swillens A., Vermeersch S., Mahieu D., Rietzschel E., De Buyzere M., Van Bortel L. (2009). Limitations and pitfalls of non-invasive measurement of arterial pressure wave reflections and pulse wave velocity. Artery Res..

[30] Kaneko K., Takahashi T., Saito H., Kiribayashi N., Omi K., Sasaki T., Niizeki T., Sugawara S. (2014). Assessment of Aortic Arch Stiffness Using Pulse-Wave Tissue Doppler Imaging: A Transesophageal Echocardiographic Comparison Study of Acute Ischemic Stroke Patients and Stroke-Free Patients. Echocardiography.

[31] Yildirim A., Genc O., Sezici E., Koca F., Tras G., Arslan A.S., Pacaci E., Ardic M.L., Alici G., Coskun M. (2025). Assessment of Aortic Arch Wall Motion Velocities in Severe Aortic Stenosis: A Transthoracic and Transesophageal Echocardiography Study. Echocardiography.

[32] Bilo G., Grillo A., Guida V., Parati G. (2018). Morning blood pressure surge: Pathophysiology, clinical relevance and therapeutic aspects. Integr. Blood Press. Control.

[33] Shimizu M., Ishikawa J., Yano Y., Hoshide S., Shimada K., Kario K. (2011). The relationship between the morning blood pressure surge and low-grade inflammation on silent cerebral infarct and clinical stroke events. Atherosclerosis.

[34] Kario K., Pickering T.G., Umeda Y., Hoshide S., Hoshide Y., Morinari M., Murata M., Kuroda T., Schwartz J.E., Shimada K. (2003). Morning surge in blood pressure as a predictor of silent and clinical cerebrovascular disease in elderly hypertensives: A prospective study. Circulation.

[35] Mavrogeni P., Molnár A., Molnár V., Tamás L., Maihoub S. (2024). Correlation Between the Pitch and Loudness of Tinnitus, Hearing Levels, and Tinnitus Handicap Inventory Scores in Patients with Chronic Subjective Tinnitus. J. Clin. Med..

[36] Patil J.D., Alrashid M.A., Eltabbakh A., Fredericks S. (2023). The association between stress, emotional states, and tinnitus: A mini-review. Front. Aging Neurosci..

[37] Jiang Y., Liu Q., Ding Y., Sun Y. (2025). Systematic review and meta-analysis of the correlation between tinnitus and mental health. Am. J. Otolaryngol..

[38] Ghalichi F., Deng X., De Champlain A., Douville Y., King M., Guidoin R. (1998). Low Reynolds number turbulence modeling of blood flow in arterial stenoses. Biorheology.

[39] Sismanis A. (2011). Pulsatile tinnitus: Contemporary assessment and management. Curr. Opin. Otolaryngol. Head Neck Surg..

[40] Klabunde R.E. (2018). Cardiovascular Physiology Concepts: Turbulent Flow.

[41] Waldvogel D., Mattle H.P., Sturzenegger M., Schroth G. (1998). Pulsatile tinnitus—A review of 84 patients. J. Neurol..

[42] Sunwoo W., Lee D.Y., Lee J.Y., Lee M., Kang Y., Park M.-H., Kim Y.H. (2018). Characteristics of tinnitus found in anemia patients and analysis of population-based survey. Auris Nasus Larynx.

[43] Lockwood A.H., Salvi R.J., Burkard R.F. (2002). Tinnitus. N. Engl. J. Med..

[44] Tomiyama M., Horio T., Yoshii M., Takiuchi S., Kamide K., Nakamura S., Yoshihara F., Nakahama H., Inenaga T., Kawano Y. (2006). Masked hypertension and target organ damage in treated hypertensive patients. Am. J. Hypertens..

[45] Borg E., Møller A.R. (1978). Noise and blood pressure: Effect of lifelong exposure in the rat. Acta Physiol. Scand..

[46] Sismanis A. (1998). Pulsatile tinnitus. A 15-year experience. Am. J. Otol..

