# Supplementary figures and images for: Association Between Morning Blood Pressure Surge and Tinnitus in Hypertensive Patients: A Cross-Sectional Study
Source: Medicina (Kaunas). 2026 Mar 10;62(3):509. doi: 10.3390/medicina62030509 (PMC13027475; doi:10.3390/medicina62030509)

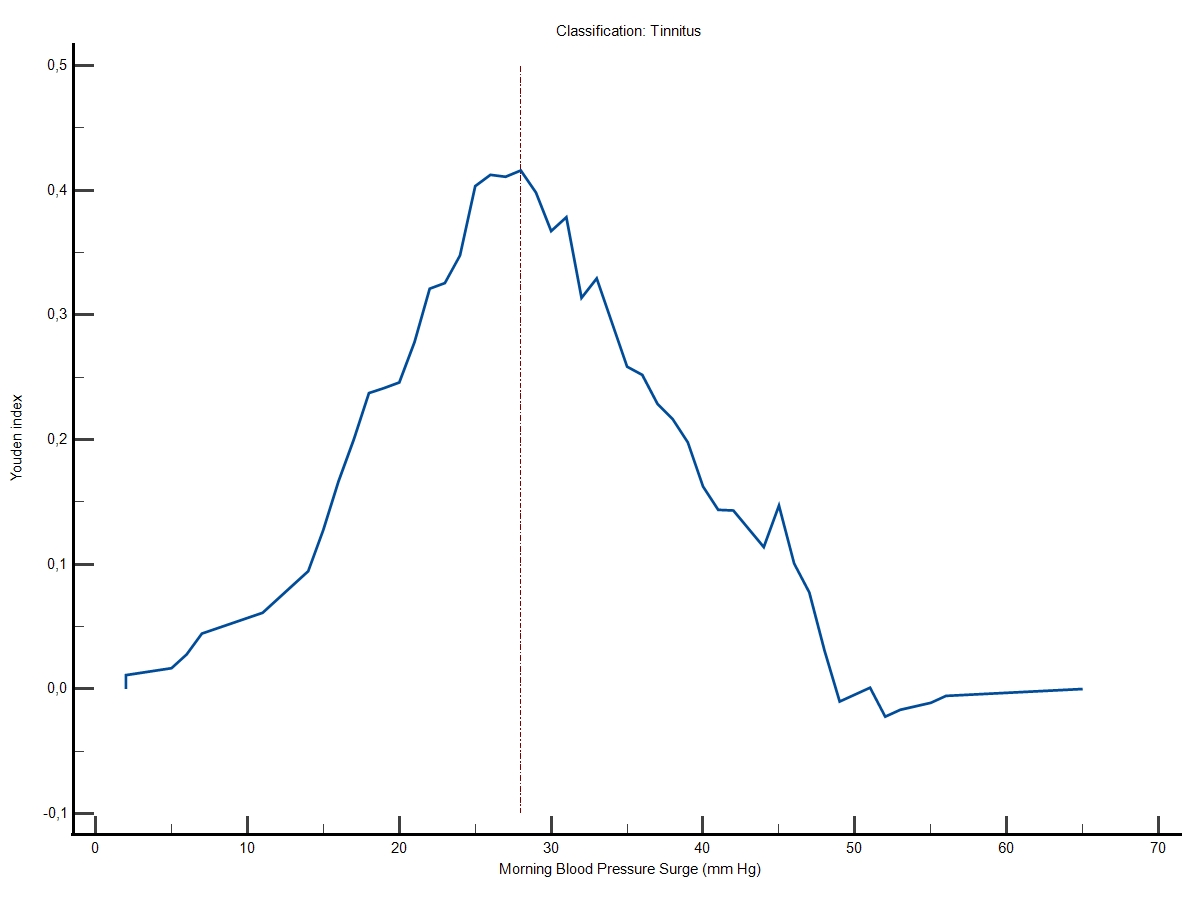

Supplement: Supplementary file 1 [file medicina-62-00509-s001.zip › medicina-4104757-supplementary.jpg]
